# Saliva. Its Chemical Constitution and Physiological Function

**Published:** 1853-04

**Authors:** A. Snowden Piggot

**Affiliations:** Professor of Anatomy and Physiology, Washington University.


AMERICAN JOURNAL

OF

DENTAL SCIENCE.

Vol. III. NEW SERIES—APRIL, 1853. NO. 3.

### ARTICLE I.

*Saliva. Its Chemical Constitution and Physiological Function.* By A. Snowden Piggot, M. D., Professor of Anatomy and Physiology, Washington University.

The importance of a thorough knowledge of the fluids of the mouth can hardly be overrated. They, of necessity, mingle themselves with all our ingesta, and exert, in this way, a powerful influence over digestion. Some of them hold important physiological relations with this function, and all of them may, in disease, materially interfere with its proper performance. None of them has a higher claim upon the attention of the physiologist or the pathologist than the saliva, which it is our purpose to examine in the present article.

The name *saliva* has been as copious a theme for debate among the etymologists as the substance itself has been among the chemists. Some have derived it from *sal*, salt, because of the abundance of salts it contains, and for a variety of other reasons which it would require too much time to particularize. Others will have it to be a corruption of σαλευω, and it certainly is a degradation of this word, which was originally applied to the roll of the yesty waves of a troubled sea. Others take it from *salvando*, because it was thought to *save* life by curing a variety of diseases, and others again from *saliendo*, because it *leaps* into the mouth from many little tubes like the jets from a fountain. Some again think it a corruption of the Greek σιαλον, which is itself involved in similar etymological difficulties. Dr. Samuel Wright, of Edinburgh, is particularly learned upon this subject, and to his admirable papers in the Lancet, which have been very freely used in the compilation of this article, we refer the reader who is inquisitive about this little piece of etymology.

This name has been applied indiscriminately to the secretions furnished by the various glands which discharge their contents into the cavity of the mouth, the parotid, the submaxillary and the sublingual, though we shall presently have occasion to show that these different organs do not produce a homogeneous liquid. The common saliva, besides being thus made up of different fluids, is mingled also with the buccal, lingual and labial mucus. Various methods have been devised to obtain it perfectly pure. The most certain of these is probably to procure it from patients suffering under salivary fistula, though it has been doubted whether this can be taken as a normal secretion. Wright's plan was, first to rinse the mouth with cold water; then, having depressed the lower jaw, to tickle the fauces so as to excite nausea without vomiting. This brings it out in a full stream, unmixed, as he thinks, with any foreign matter. The admixture of extraneous substances with the liquid thus obtained, cannot indeed be great, but that some mucus must get access to it is manifest upon a moment's reflection. Epithelium scales and mucus from the ducts must always be present, in whatever way it may be obtained, and to this we probably owe the globules which the microscope detects in such quantities in the saliva. The time at which this fluid is obtained for examination, is by no means unimportant. Wright suggests that it be collected from a healthy person after a fast of about three hours.

Healthy saliva is described by the last named observer as a "limpid fluid, having a faint blue tinge and a slight degree of viscidity. It is perfectly uniform in consistence and unobscured by frothiness or flocculi. It possesses a faint sickly odor, *sui generis*, due to its constituent, *ptyalin;* this odor is strengthened by heat and by most acids, but alkalies diminish and destroy it." Most observers assert that it is always totally tasteless, but Wright insists that it has a manifest sapor. He acknowledges that, to the individual secreting it, the freshly formed fluid is tasteless, but asserts that the saliva of another is always sapid, and that a man may retain his own in his mouth until it shall possess taste which becomes very distinct if it is collected in a vessel and then applied to the tongue. Lehmann and Jacubowitsch, the most recent writers on this subject, are at variance with Wright upon this point, and suggest that the saliva be experimented on may have been a little stale, a matter of some consequence in so readily decomposable a fluid.

The *quantity* of this fluid which is secreted in the course of a day, has never been exactly ascertained and must be subject to great variation. The quantity of fluid ingested, the amount of motion in the muscles of mastication, the character of the substances taken as food, all must influence it. Movements of the jaw, stimulating condiments, even the thought of food increases its flow. Mitscherlich obtained from a patient laboring under salivary fistula in the duct of Steno about 2½ ounces in twenty-four hours. At the same time he ascertained that the whole amount secreted was about six times the product of this single gland. It is probable, therefore, that this patient was making about 20 ounces troy of saliva in twenty-four hours.

The *specific gravity* of saliva is another point upon which observers are not unanimous.* The discrepancies here are very great, and are partly due, undoubtedly to the greater or less admixture of mucus with the fluid tested by different authors. Wright's experiments, however, which are entitled to the utmost

*The following are some of the estimates of different authors: Haller states it at 1045 (water being 1000;) Lamure at 1119; Siebold at 1080; Thomson at 1003.8; Tiedemann and Gmelin at 1004.3; Mitscherlich at 1006.1 and 1008.8; Golding Bird at 1009.1 and Wright at 1007.9. Lehmann reckons it at from 1004 to 1006, and says it may rise during health to 1009 or sink to 1002. Jacubowitsch makes it 1002.6 before filtration and 1002.3 afterwards.

respect from their number, having been made upon more than five thousand individuals, and from the accuracy and candor of their author, show that, even in perfect health, the density of saliva varies greatly, in consequence of idiosyncracy, the nature of the ingesta and a variety of other circumstances. He found it always denser after a meal than before it, in the evening than early in the day. It is "commonly thickened by an abundant use of animal diet, by fatty food especially and by oily fish. Oysters and vegetable diet produce an opposite effect. I tried the specific gravity of the saliva in a healthy man for a week, and found its extremes to be 1007.9 and 1008.5. I then kept him upon vegetable diet and water for a week, during which time the lowest sp. gr. of his saliva was 1003.9, and the highest 1004.7. Throughout the following week he took nothing but animal food and water, with four ounces of bread daily; and the extremes of the sp. gr. of his saliva were 1009.8 and 1017.6. All alcoholic stimulants have a tendency to thicken the saliva, and in large quantities they not only alter its consistence but materially diminish its activity. Moral emotions, variations in the state of the weather, electrical conditions of the atmosphere, light, sound, and other contingent circumstances, exert a remarkable influence upon the secretion of saliva and also upon its specific gravity. Hence the difficulty of making accurate observations concerning it."

Its reaction has also been a subject of discussion. Wright believes that alkalinity is essential to the proper performance of the physiological function of the saliva. He found the alkali to vary from .095 to .303 per cent., a proportion which may, however, be considerably increased. If, at any time, it should exceed one per cent., he regards as an evidence of disease.*

* From Wright's method of stating this, and from the context, it is manifest that he supposes the alkaline reaction of the saliva to depend upon the presence of free soda. Now as it requires about 1.28 of sulphuric acid to neutralize one part of soda, we can easily make the statement in a manner which shall express the facts without committing us to any theory. This would be that it requires usually from .122 to .388 of sulphuric acid to neutralize 100 of saliva, and should this amount increase to 1.28, we may suspect disease.

He observed a remarkable connexion between this secretion and the semen, a fact of no little interest, if we connect with it the well known pathological sympathy between the parotid gland and the testicles in mumps. After *coitus* in dogs the alkalinity of the saliva was notably diminished, and in both man and animals it is generally increased by abstinence from sexual intercourse. It is also increased during digestion and diminished in fasting. In the latter state, it sometimes becomes acid, especially if the abstinence be protracted, but, in moderate fasting (e. g. from 6 to 12 hours,) Wright says the fluid may become neutral but should never be acid. When, from admixture of mucus or other causes, it exhibits a reaction of this character, he suggests that some spirit or pepper be taken in the mouth, under the stimulus of which, in a *healthy person*, the quantity of alkali is always very much increased. He says he has known the proportion of alkali to be increased, during the space of a quarter of an hour from .2 to 1.9 per cent. by the local application of an irritant. Slowness of digestion from the presence of much fatty matter, alcohol or vinegar in the stomach is, in like manner, accompanied by a decided increase in the quantity of alkali. Frerichs found that in a man smoking tobacco, the quantity of alkali was so much diminished that it required but .15 gramme sulphuric acid to neutralize 100 grammes of saliva.

Mitscherlich was the first to isolate the saliva of one of the glands, he examined the fluid collected from the parotid at a fistulous opening in the duct of Steno. Since his time it has been examined by Van Setten and Garrod; and Magendie and Claude Bernard have investigated the fluids of the different larger glands, which they obtained from their own persons, by introducing tubes into the different ducts. From these experiments it is manifest that the secretions furnished by the different glands, differ widely from one another in chemical composition and in physiological use. That from the parotid and sublingual is clear, limpid and thin, while that from the submaxillary is thick and viscid, resembling simple syrup both in color and consistence.

The specific gravity of the *parotid saliva* is stated by Golding Bird at 1007.5. In dogs, Jacubowitsch found it to vary from 1004 to 1004.7; in horses, according to Lehmann it ranges from 1005.1 to 1007.4.

Its reaction is usually alkaline during a meal, acid when fasting. This statement which was first made by Mitscherlich has been confirmed by Marshall and Garrod. These latter observers are inclined to attribute this to the rapidity of the discharge during a meal, and the slowness with which the secretion is formed at other times. They found that ordinarily but two or three drops were discharged by one parotid gland in a quarter of an hour and that this was acid, but that in half a minute after a morsel had been taken into the mouth, the reaction was neutral, and within the minute, alkaline. This continued until twenty minutes after the meal, when it again became acid. This is accounted for by the fact of the general acidity of the mucus surfaces of the mouth, which is sufficient to overpower the feeble alkalibility of the saliva when secreted in small quantity. When the flow, however, is increased, the saliva more than neutralizes the mucus, and hence we have the alkaline reaction. The mucus, in cases of parotid fistula, comes, of course, from the lining membrane of the ducts.*

Upon what does this reaction depend? Shultz supposed it to be caused by ammonia. This is, however, impossiblc, because the fluid distilled from saliva is not alkaline, because saliva evaporated at a high temperature is increased and not diminished in alkalinity and because test papers discolored by it do not regain their original tint when heated. These phenomena are incompatible with the hypothesis of a volatile alkali. Wright, Garrod and others supposed it to depend on free soda, mainly because potassa could not be detected in it. The opinion, first broached we believe by G. Owen Rees, in his paper on Saliva, in the Cyclopœdia of Anatomy and Physiology, that the tribasic phosphate of soda is the cause of this reaction, appears

* Mitscherlich found that it required .223 of sulphuric acid to naturalize 100 of parotid saliva.

to be gaining ground. Strength is given to it by the generally acknowledged fact that the same salt communicates alkalinity to serum.

The composition of parotid saliva does not differ much from that of the common fluid. Like the latter, it contains *ptyalin*, extractive matter, sulphocyanide of potassium, epithelium and mucous corpuscles, a volatile acid of the butyric group (probably caproic) combined with potassa, chlorides of sodium and potassium, a small amount of phosphates and a trace of alkaline sulphates.

The saliva of the *submaxillary gland*, according to Jacubowitsch has a specific gravity of 1004.1, a less strong alkaline reaction and less combined organic matter than parotid saliva.

We return now to the chemistry of the common saliva which has been oftener and more thoroughly studied than that from the separate glands. One of the most characteristic of its organic constituents and one of the most important to the due physiological action of this fluid is that known as *ptyalin*. Unfortunately, however, the greatest confusion prevails among chemists in their statements of the nature, composition and reactions of this substance. Upon a careful examination of their modes of obtaining it, it becomes manifest that they have been dealing with different substances, and consequently there must be discrepancies in their accounts of the physical and chemical character of ptyalin. This will be plain from a short review of the various descriptions they have left us of their manipulations and experiments.

Berzelius first separated from saliva a substance to which he gave the name of salivary matter or ptyalin. He first evaporated the saliva to dryness, and then exhausted it with alcohol, thus removing the osmazome, fat, chlorides and lactates. The solid residue, being alkaline, was neutralized with acetic acid and the acetate removed by fresh alcohol. What remained, being dried, was exhausted by water which took up this ptyalin and left behind mucus with earthy sulphates and phosphates. "The solution of this matter in water," says Berzelius, "is a little consistent and is not troubled by ebullition. After evaporation, the salivary matter remains colorless and transparent. If water is then poured upon this last, it becomes at first white, opaque and mucus, and then dissolves, making a clear solution which is not precipitated by tincture of galls, chloride of mercury, subacetate of lead nor the strong acids, characters which distinguish this substance from a great number of other animal matters."

Dr. Golding Bird, in reviewing the characteristics of *ptyalin* as described by previous chemists,* comes to the conclusion that it does not exist as a distinct principle. His reasons for this are, first, the discrepancy in the accounts given of the substance by different observers; secondly, the constant formation of new insoluble matter, every time the solution is evaporated, a point noticed by Gmelin, Mitscherlich and Schultz. This character certainly approximates this substance to the albuminate of soda. Dr. Bird's own experiments exhibit still further this analogy. He prepared some *ptyalin*, according to the process of Berzelius, and then exposed it to the action of a *couronne de tasses*, of thirty-six pairs. "Coagulation ensued at both electrodes, but most copiously at the negative, when an odor of chlorine was evolved; and by no character whatever could it be distinguished from albumen."

Simon's method of obtaining this substance is analogous, but not entirely similar to Berzelius. A known weight of saliva was evaporated to dryness; the loss of weight thus indicated the proportion of water. The residue was treated with ether, which extracted the fats. The solid mass remaining was next treated with water, which dissolved out the ptyalin, extractive matter and salts, leaving behind mucus, albumen and cells. Evaporated to a small bulk, the fluid gives up its ptyalin as a precipitate on the addition of alcohol. This method alone is a sufficient refutation of Dr. Bird's hypothesis, the heat used in the process being high enough to coagulate all the albumen contained in the saliva.

Lehmann says that it is obtained in greatest purity from the

* London Med. Gazette, 1840.

spirit-extract of saliva, after repeated extraction with alcohol and ether. It is combined with potash, soda and lime, which may be separated from it by carbonic or some stronger acid, after which it dissolves, though with difficulty, in water. When separated by the acids, it falls in amorphous flocculi; which, as already said, are difficult of solution in pure water but dissolve readily in water to which either an alkali or an acid has been added.

Its reactions, as stated by the latter chemist, sufficiently distinguish it from albumen, while they show it to be intimately related to that substance. *Acetic acid*, added to its alkaline solution, throws down a flocculent precipitate which readily dissolves in an excess of the precipitant. Boiled with *chloride of ammonium* or *sulphate of magnesia* the alkaline solution of ptyaline becomes very turbid. The alkaline solution is precipitated by *tannic acid*, *chloride of mercury* and *basic acetate of lead*, but not by *alum*, *sulphate of copper*, *&c.* The acetic acid solution is strongly precipitated by *ferrocyanite of potassium*, and when boiled with *nitric acid*, it yields a yellow color. The resemblance, it will be observed, is much more striking between this substance and albuminose or the modified albumen of Mialhe, than between it and normal albumen. Both these substances, it may be remarked, are albumen, in a state of change, entering the economy. The physiological analogies between ptyalin and the other digestive ferments will presently be alluded to. When these are all considered, it would appear as if this were the result of the waste of some of the tissues of the glands, probably of the mucous membrane of the ducts and their ramifications, still undergoing the process of metamorphosis, and therefore acting as a ferment.

The *ptyalin* of Dr. Wright must not be confounded with the substance we have just described. It manifestly belongs to the class of fats. The most cursory examination of his experiments suffices to convince us of this.

The following are the means employed by him for preparing a pure specimen of ptyalin: "First, to pass saliva through ordinary filtering paper, and, after filtration shall have been completed; secondly, to exhaust the residue with sulphuric ether; the ethereal solution contains a fatty acid and ptyalin. It is to be allowed to evaporate spontaneously; and thirdly, the residue left by evaporation is to be placed upon a filter, and acted upon by distilled water which dissolves the ptyalin and leaves the fatty acid. If the aqueous solution be carefully evaporated to dryness, the 'salivary matter' will be obtained in a pure state. Ptyalin, as thus prepared, is a yellowish-white, adhesive and nearly solid matter, neither acid nor alkaline, readily soluble in ether, alcohol and essential oils, and more sparingly soluble in water. It alone possesses the characteristic odor of saliva; it is unaffected by galvanism and most of the agents which coagulate albumen; it is abundantly precipitated by subacetate of lead and nitrate of silver; feebly so by acetate and nitrate of lead and tincture of galls, uninfluenced by bichloride of mercury and strong acids; the latter considerably heighten its proper odor and impair its solubility, whilst alkalies render it more soluble, and give it the smell of mucus. Moderate heat and oxygen gas also increase its odor; but intense heat or cold diminishes or entirely destroys it. At a suitable temperature, ptyalin may be preserved for any length of time without risk of decomposition. The salivary fluid from which ptyalin has been removed by filtration, possesses a sickly mucous smell, decomposes much sooner than ordinary saliva, and in the process of decay invariably evolves ammonia. If this fluid be heated, the mucous smell will be increased until the evaporation shall have been continued nearly to dryness, when a slight salivary odor may be recognized, due to a portion of ptyalin being liberated from the mucus with which it was previously in combination."

The other constituents of saliva are, extractive matter soluble in alcohol and water, precipitable by tannic acid but not by alum; sulphocyanide of potassium; caproate of potassa;* epithelium and mucus-corpuscles; chlorides of sodium, potassium

* Probably. It is according to Lehmann, the potash-salt of a not very volatile acid of the butyric acid group. The crystals under the microscope resembled tufts of margaric acid.

and calcium; a small amount of phosphates; traces of sulphates, lactates and carbonates. Lehmann says that the quantity of the latter salts in the fresh secretion must be extremely small and that the alkaline carbonates which have been observed are formed during the process of analysis from the action of the atmospheric air. The formation of carbonate of lime, in this manner, in the parotid secretion of the horse, may be easily seen—very beautiful crystals of this salt being formed.

"In the parotid saliva of the dog, the solid residue, according to Jacubowitsch, amounts to 0.47 per cent., in which the organic matter is to the inorganic as 29.8, 70.2, the latter consisting of 44.7 of alkaline chlorides and 25.5 of carbonate of lime. In the corresponding fluid of the horse, Lehmann found, as the mean of six analyses, 0.708 per cent. of solid residue, in which the organic was to the inorganic matter as 46.1, 53.9. The mean amount of ptyalin was 0.140 in Lehmann's experiments."

The presence of sulphocyanogen, in the saliva, was for a long time a subject of quite earnest discussion. At last the question is settled in favor of those who believe it to be a constant ingredient in this secretion. Whatever may be its physiological value, it is a matter of great importance in a medico-legal point of view, to determine whether it is a normal constituent of saliva. For, in a state of sufficient concentration, it strikes with the sesquichloride of iron the same blood-red tint as meconic acid.

Treviranus was the first to call attention to this reaction. It is unnecessary to go into the history of the discussion upon this point, since the question has been already settled. It will suffice to mention the various modes of proving the presence of sulphocyanogen. Dr. Golding Bird's plan is to acidulate the saliva with nitric acid, mingled with chloride of barium and then to filter. "No change will occur till the mixture be warmed, when sulphuric acid will be formed at the expense of the sulphocyanogen, and a copious precipitate of sulphate of barytes will be formed." Dr. Percy advises that the residue of carefully dried saliva be exhausted with alcohol and the solution be subjected, in a test tube, to the action of nascent hydrogen, which, at the moment of its escape, decomposes the sulphocyanogen, generating sulphuretted hydrogen, which may be recognized by its smell and its action upon lead paper. A piece of pure zinc, with sulphuric acid, is a convenient substance for generating the hydrogen. Still further the red precipitate with the per salt of iron may be easily tested. If it is a sulphocyanide, heat will temporarily destroy its color; a reaction which distinguishes it equally from the meconates, formiates and acetates. Another simple and easy method of recognizing the precipitate from a solution containing sulphocyanogen, is to drop a crystal of corrosive sublimate in the red fluid. If the tint be produced by sulphocyanide, the color vanishes, if by any of the acids alluded to, it is unchanged.

There are two modes in which the quantitative determination of this substance may be made. The alcoholic extract may be dissolved in water; the solution filtered to free it from fat; the filtrate concentrated, treated with sulphuric acid and distilled. The distillate must then be saturated with baryta and filtered, and the filtered fluid evaporated. The residue is to be boiled with fuming nitric acid or aqua regia, and the amount of sulphocyanogen calculated from the sulphate of baryta which separates. The other method is, to precipitate the same aqueous solution with nitrate of silver, to wash the precipitate well, to treat it with water containing nitric acid which does not dissolve the chloride of silver, the silver is precipitated from the acid solution by hydrochloric acid, a little chloride of barium is added, the solution is evaporated, a little nitric acid being repeatedly added. We thus, also, obtain sulphate of baryta, from which the sulphocyanogen is to be calculated. It must be borne in mind, however, in all calculations of this kind, that, according to Dr. Wright, this ingredient of the saliva is temporarily augmented by any local stimulation of the glands, by the internal use of prussic acid, the cyanides and sulphur, especially by the last named substance.

After dilution with water, saliva lets fall a flaky precipitate, which is dissolved by boiling and deposited again on cooling.

Saliva freezes at a lower temperature than water. Wright says he has never seen the healthy liquid resist 10° F. though in disease it may remain fluid at 0°.

Saliva has a strong affinity for oxygen, absorbs it readily from the air and imparts it to other bodies. It has been even said to oxidate gold and silver when in minute division they are triturated with it. The latter metal is undoubtedly affected by it. In manufacturing mercurial ointment, it has long been known that the globules are broken down more readily when the operator spits in the mortar, and the oxidizable metals are always more corroded by this fluid than by pure water. Pure saliva, according to Wright, absorbs, on an average, its own volume of oxygen, though this is liable to variation, from two and a quarter times to one-half the bulk of the secretion. This difference is supposed to be due to the carbonic acid gas contained in the secretion, which varies from one-eighth to one-twelfth in volume, though it is sometimes more abundant. This gas is absorbed in the same ratio, but hydrogen and nitrogen in less proportion.

Exposed to the air, at a temperature of 60° F., saliva readily putrefies. Decomposition commences at from the third to the seventh day, the ptyalin generally being the first ingredient to suffer. "Ammonia is usually formed during the process of decay; but, if the saliva have been previously heated to 212° F., it decomposes very slowly, and the product of destruction is generally an acid. The addition of an acid to saliva also assists in its preservation ; whilst caustic alkali, which almost immediately causes the evolution of ammonia, promotes rapid decomposition. If saliva be carefully evaporated to dryness, it will retain its odor and properties in an unimpaired state for many months."

The destructive distillation of this secretion produces, according to the same accurate observer we have so often quoted, carbonate of ammonia, oily matter, acetic and lactic acids but never the hydrocyanic. The residue consists chiefly of phosphates and chlorides with a little carbonate.

The *analysis* of saliva, has been conducted in different ways by different chemists. Wright's method was as follows:

"The saliva is to be filtered through moderately coarse filtering-paper. On the filter will remain a solid residuum (1,) and a clear liquid (2) will have passed through.

*Examination of solid residuum* (1.) It is to be washed thoroughly on the filter with ether, when a residue (A) will be left, and an ethereal solution (B) will be obtained.

*Examination of* (A.) Exhaust with cold distilled water; this dissolves the chlorides of sodium and potassium (*a*, *b*) which may be obtained by evaporation. The matter left upon the filter is to be dried, weighed and then incinerated. The amount of loss thus occasioned indicates the proportion of free albumen (*c*.) A little ash remains from which distilled water extracts carbonate of soda (*d*) and leaves phosphate of lime (*e*.)

*Examination of* (B.) Evaporate carefully to dryness. Wash the residuum on a filter with distilled water and continue to treat with this menstruum until every thing soluble in it is removed. The aqueous solution is next to be evaporated to dryness, when a residue of pure ptyalin (*f*) will be obtained. The matter left upon the filter is a fatty acid (*g*) which is to be dissolved in sulphuric ether, from which, by evaporation, it may be obtained in a pure form.

*Examination of filtered liquid* (2.) This liquid is affected neither by boiling nor by nitric acid; yet it contains albuminate of soda (*h*,) which can only be separated by means of galvanism. To this end, it is advisable to reduce the liquid by very careful evaporation, one-third in volume, and then to introduce the wires of a galvanic battery in action: free soda will collect upon the negative pole, and coagulated albumen upon the positive one. In this manner all the albuminate of soda may be removed without decomposing the chlorides.

The liquid having been thus treated, is next to be very carefully evaporated to dryness, and the dry residuum exhausted with ether, which dissolves the lactates of potassa and soda (*i*, *j*,) and sulphocyanide of potassium (*k*.) These salts, having been dried and weighed, are to be dissolved in distilled water, and subacetate of lead is to be added to the solution until it shall cease to afford a precipitate. An insoluble sulphocyanide of lead will be deposited and a soluble lactate will remain in solution. The former, after having been dried and weighed, will indicate the proportion of sulphocyanide of potassium previously existing in the saliva; and by subtracting this weight from that of the original saline matter, the quantity of lactates will also be determined.

The residuum from which these salts have been separated is to be treated with alcohol, which will remove the remaining chlorides of sodium and potassium, the weight of which is to be added to that of (*a*, *b*.)

After the extraction of the chlorides, the soda, which remains in the form of carbonate, is to be neutralized by acetic acid, and the salt dissolved out by alcohol: by evaporating the alcoholic solution to dryness, and exposing the salt to a red heat, the acetic acid will be destroyed, and the soda (*l*) left.

The remaining solid matter is to be dried, weighed and incinerated. The loss by incineration determines the amount of mucus (*m*.)

The saline matter left is to be boiled in distilled water, which will generally remove a little sulphate or phosphate of potassa or soda—usually a phosphate of soda (*n*.)

The last residuum will be a phosphate of lime: its weight must be added to that of (*e*.)

Silica is mentioned as a constituent of saliva. I have never met with it, though I have occasionally discovered a trace of iron."

Simon's plan differs somewhat from this. He evaporated a known quantity to dryness and thus determined the water. He then treated the residue with ether to extract the fats; and with water, to take up the ptyalin, extractive and salts. "The insoluble residue that had resisted the action of ether and water, consisted of albumen and mucus. Another portion of the saliva was decanted from its precipitate, evaporated to a small residue, and the ptyalin, with a trace of extractive matter, precipitated by alcohol. When the saliva contains a caseous matter, (which I have observed in large quantity in the saliva of the horse,) the precipitate of ptyalin and casein produced by the alcohol must be dissolved in water, and the casein then thrown down by the careful addition of acetic acid. In this case, a portion of the casein precipitated by the alcohol usually remains undissolved by the water. I have detected free acetic acid in the saliva discharged during salivation. In order to determine its quantity, the saliva must be accurately neutralized by a solution of carbonate of potash of known strength; from the amount of the alkaline solution required, the quantity of acetic acid can be calculated. If, in addition to acetic acid, free lactic acid is likewise present, the residue of the saliva, after evaporation, when dissolved in water, will still indicate an acid reaction, because lactic acid differs from acetic acid in not being volatilized at the ordinary temperature used for evaporating animal fluids. In order to determine the amount of free soda in the saliva, the dried residue must be extracted with alcohol; the free soda (which is left in the residue) must be saturated with acetic acid, the resulting acetate of soda extracted with alcohol, evaporated, and by incineration, reduced to carbonate of soda."

The following are the results obtained by some of the most distinguished observers.

Berzelius found in 1000 parts of human saliva,

| | |
|---|---|
| Water, | 992.9 |
| Ptyalin, | 2.9 |
| Mucus, | 1.4 |
| Extract of flesh with alkaline lactates, | .9 |
| Chloride of sodium, | 1.7 |
| Soda, | .2 |

The result obtained by Simon from his own saliva was,

| | | |
|---|---|---|
| Water, | | 991.225 |
| Solid constituents, | | |
| Fat, containing cholesterine, | .525 | |
| Ptyalin, with extractive matter, | 4.375 | |
| Extractive matter and salts, | 2.450 | |
| Albumen, mucus and cells, | 1.400 | 8.775—1000. |

Wright's analysis, in which, it must be remembered, the *ptyalin* differs from that of Berzelius and Simon, gave

| | |
|---|---|
| Water, | 988.1 |
| *Ptyalin,* | 1.8 |
| Fatty acid, | .5 |
| Chlorides of sodium and potassium, | 1.4 |
| Albumen with soda, | .9 |
| Phosphate of lime, | .6 |
| Albuminate of soda, | .8 |
| Lactates of potash and soda, | .7 |
| Sulphocyanide of potassium, | .9 |
| Soda, | .5 |
| Mucus, with ptyalin, | 2.6 |

The analysis, published by Frerichs and copied by Carpenter in the last edition of his Principles of Human Physiology, from which we quote, does not precisely correspond with either of these others:

| | | |
|---|---|---|
| Water, | | 994.10 |
| Solid matters, | | |
| Ptyalin, | 1.41 | |
| Mucus and epithelium, | 2.13 | |
| Fatty matter, | .07 | |
| Sulphocyanide of potassium, | .10 | |
| Alkaline and earthy chlorides and phosphates, and oxide of iron, | 2.19 | 5.90 |
| | | 1000.00 |

In regard to these analyses, Wright says he made about twenty of healthy saliva and that no two of them *exactly* corresponded. The variation between Frerichs and Wright is especially manifest in the estimation of the sulphocyanide of potassium. Both of them are beyond Jacubowitsch and Lehmann. The first of these chemists found 0.06 parts of this salt in a thousand of his own saliva, and the second states its variation at 0.046 to 0.089. Frerichs' advance upon the average of the last is but trifling.

In regard to the elimination of substances taken into the system, through the medium of this secretion, Lehmann shows that many of them pass off more rapidly by the salivary glands than by the kidneys. Thus, iodide of potassium being taken in the form of pill, iodine may be detected in the saliva in ten minutes, while it requires from half an hour to two hours, to detect the same in the kidneys. Bromine, mercury and other sialagogues obey the same law. Lehmann, like many of the earlier observers, found mercury in the saliva discharged during hydrargyria. We shall, however, return to this theme again.

The physiological use of saliva has long been a subject of debate among the learned, and cannot yet be regarded as fully and satisfactorily ascertained. Dr. Wright, in commencing his investigations in reference to this particular, first endeavored to ascertain the influence upon vegetable and animal life. We have no room even for an abstract of his extremely interesting experiments, and refer our readers who desire information on this subject, to his papers in the Lancet for 1842, and to the British and Foreign Medical Review for January, 1847. Suffice it to say, that vegetables were injuriously affected by it, while their seeds escaped, and that animals were destroyed by it when their veins were injected with it. Dogs manifested unequivocal signs of hydrophobia, in several instances, and, in nearly all, perished with symptoms of great disturbance of the nervous system. Jacubowitsch and Lehmann both deny that saliva exerts this deleterious influence over either plants or animals.

Pringle's experiments are not to be neglected by the student who wishes to get an idea of the influence which this fluid exerts over the digestive processes. He beat up in a mortar two drachms of fresh meat, the same quantity of bread, with an ounce of water and so much saliva as he thought necessary for digestion; put the mixture in a closed phial and set it in a furnace. It remained two days without any visible fermentation, but, on the third, the bread and flesh had risen in the water, a sediment was forming and bubbles of air continually escaping, this action being accompanied by the vinous smell common to fermenting liquors. When completed, the fluid had a pure acid taste, without any putrid smell, from which, indeed, it had been free during the whole time of the experiment. He thought that healthy saliva was "qualified for retarding putrefaction, preventing immoderate fermentation, acidity and flatulence in the *primæ viæ*." A subsequent experiment with putrid saliva, showed that this liquid in that state brought on fermentation sooner, made the flesh unusually putrid, but, at last, in consequence of the acid generated, removed the offensive odor.

Leuchs is usually supposed to be the first to discover that saliva had the property of converting starch into sugar, but Dr. Wright has expended much learned research to show that this action upon starch was known long before the announcement of that chemist's experiments in 1831. He quotes Boerhaave, Plenck, Mundius and a host of others, to show that the influence of saliva in producing a saccharine fermentation in farinaceous liquids was well known to the older writers. The favorite drink of the American savages, according to the statements of these authors, was formed by beating up maize, cassava, or some other amylaceous substance with water, into which were thrown, from time to time, fragments of the same substance which, having been chewed by the women, were thoroughly saturated with saliva, and so acted as a ferment, inducing the desired degree of saccharine change in the mass. Schwann corroborated the statement of Leuchs by his experiments. He found that when boiled starch was digested for twenty-four hours in saliva, it no longer gave a blue tint on the addition of iodine; on neutralizing, drying and digesting in alcohol, he obtained sugar, recognized by its taste and its property of fermenting with yeast. The residue, which alcohol did not dissolve, was found to be salivary matter and starch altered to a substance resembling gum, with which iodine did not produce a blue tint.

Wright has investigated this subject with his usual accuracy and candor. He ascertained, as the result of a number of experiments that the production of sugar from starch is a constant result of the action of saliva upon the latter substance. Fresh saliva seemed to have the most powerful action, though this catalytic or zymotic power remained even after boiling. Hydrogen and nitrogen seemed to diminish the activity of the saliva. Oxygen did not appear to increase it. The addition of either acids or alkalies prevented the development of sugar in the mixed liquids.

The same observer was satisfied that this fluid exerted a digestive influence over meats. He found that on subjecting the same meat to water and to saliva at the same temperature, fermentation went on rapidly in that portion immersed in the saliva, while in that digested in water there was no perceptible change.

The most conclusive of his experiments, however, in this department of inquiry, are those performed on two dogs of about the same weight and both in perfect health. Into the stomach of one of these animals, after a fast of twenty hours, were injected eight ounces of lean, raw beef and the same quantity of bread, beaten to a pulp with ten ounces of water. The gullet was tied, and the saliva tested, and found to be alkaline. "*In half an hour its strength of alkalinity was at least doubled;* the *quantity* of the secretion was also much augmented. *The alkaline reaction of the saliva gradually increased, till, at the end of three hours, it contained* 3.14 *per cent. of alkali.*" The animal, being killed, by introduction of air into the jugular vein, the food was found unaltered, except by the addition of mucus, and the contents of the stomach had a sour smell. That viscus was deeply reddened, the tint being three or four times deeper than is customary during digestion. On the *second* dog, the same experiment was performed, differing only in the substitution of *ten ounces of alkaline saliva* for the ten ounces of water in the preceding experiment. The saliva here, too, at the beginning, was moderately alkaline. "*At the end of half an hour, the secretion was scarcely altered either in character or quantity.* When two hours had elapsed, the mouth was very very frothy, but the saliva was little changed. At the end of three hours, the mouth was still frothy, *and the saliva contained only* 89 *per cent. of alkali.* The animal being killed in the same way, the contents of the stomach were found not particularly acid, nor its mucous membrane more vascular than in healthy digestion. The food was reduced to a perfectly hemogeneous pulp."

Dr. Wright's conclusions from his experiments are

"1. Saliva has the power of modifying, and, to a certain extent, of digesting vegetable and animal substances.

2. It has a more powerful action upon vegetable than upon animal matters.

3. The healthy digestive action of saliva is always attended with the evolution of lactic acid.

4. Filtration, or boiling, diminishes the digestive powers of saliva, but does not destroy them.

5. Exposure of the saliva to atmospheric air, for a moderate length of time, does not materially weaken its digestive powers, but they are enfeebled in the ratio of the putrescency of the secretion.

6. Oxygen gas assists the digestive action of saliva, but is not essential to it. Carbonic acid gas impairs this action in a mild degree, and hydrogen and nitrogen gases weaken it very considerably.

7. Acids or alkalies, added to saliva, diminish or destroy its digestive properties.

8. The presence of saliva in the stomach is essential to healthy digestion.

9. The digestive action of saliva is not possessed, in any efficient degree, by animal mucus, acids, alkalies, or alkaline salts."

According to the same authority, all stimulation of the stomach, by alcohol, spices or acescent food, is accompanied by a marked increase in the alkalinity of the saliva. When spitting much, after a full meal, he never failed to have acidity and pain in the stomach with corresponding alkalinity of the saliva. A dose of carbonate of soda, neutralizing the gastric acidity, *brought down the saliva to its usual standard of feeble alkalinity, in a few minutes, and often in a few seconds.* Acids introduced into the stomach, raw turnips, onions and other indigestible substances, produced the same effect upon the saliva. The importance of this fluid in digestion is further shown by the dyspepsia consequent upon its waste. This is undoubtedly one of the sources of that ill-health with which inveterate chewers and smokers are sometimes afflicted. "Frequent attacks of dyspepsia, to which I was once happily a stranger," says Wright, "painfully remind me of the injury which I sustained in the course of my investigations. I once spat two hundred and fifty drachms of saliva in one week, and from the nature of my experiments I was often compelled to spit directly after dinner; in that seven days I lost eleven pounds in weight and was much weakened and emaciated."

The uses of this fluid in the economy, are divided, by the author just quoted, into active and passive; the active being, (1) stimulation of the stomach, (2) aiding digestion by a specific action upon the food, (3) neutralization of undue acidity by supplying a corresponding portion of alkali; and the passive, (1) assisting the sense of taste, (2) favoring the expression of the voice, and (3) clearing the mucous membrane of the mouth.

Cl. Bernard, the distinguished French experimentalist, has investigated recently the action of saliva in digestion, and favors Beaumont's views of the mere mechanical agency of this fluid; opinions, we think, sufficiently controverted by Wright in the experiments just quoted. Mr. Bernard, however, has certainly advanced our knowledge of saliva as will be seen by a brief review of his statements.

We have already mentioned his discovery of the difference, in physical characters, of the different secretions which make up the common saliva. He also found them to differ in their physiological action, that of the parotid and sublingual being to saturate the food, and that of the submaxillary, by its glutinous consistence, to facilitate deglutition. To confirm this opinion, he made an opening in the œsophagus of a horse, whence he drew the alimentary bolus, as it descended, and found that it had increased elevenfold by the imbibition of saliva. He next tied Wharton's (Steno's?) duct and found that the animal required forty-one minutes to masticate what before had demanded but nine minutes; and the mass, when withdrawn, was covered with mucus and a glutinous fluid, the interior being dry and friable, and the whole increased in weight only three and a half times. Water seemed to promote mastication as much as the parotidean saliva, this latter being in proportion to the dryness of the ingested substance.

As to its action upon amylaceous substances, Bernard maintains it to be very gradual, and thinks the time the food remains in the mouth to be too short for it to exert any other than a very partial transforming influence upon it, the change being, he thinks, arrested by the acidity of the gastric juice. In this statement he is contradicted by both Frerichs and Bence Jones who have shown that the action is still kept up in the stomach, notwithstanding the acidity of the gastric fluid. He killed a dog, fed on potatoes, and found in his stomach only a trace of sugar, but much unaltered starch. He agrees with Magendie in stating that neither the secretion of the parotid nor that of any other of the glands taken separately, nor yet when mixed with each other, exerts any transforming influence upon starch; and therefore assigns the source of the active principle to the small buccal glands. In this statement, he is positively contradicted by the experiments of Marshall and Garrod, who obtained the saliva on which they experimented from the parotid, through a fistulous opening on the cheek, and found it to change starch to sugar. Bernard grants that ptyalin possesses this property, but affirms that it is not peculiar to the salivary secretion, being possessed in an equal degree by many animal fluids, as the water in which buccal mucous membrane had been soaked, the serum of the blood, mucus from the nose during coryza, &c., whence he concludes that the salivary substance does not differ from other nitrogenous substances in a state of change. Here again his statements are at variance with those of Wright, who experimented with various animal fluids, among them intestinal mucus and the serum of blood, without, in any case, producing this metamorphosis.

None of these observers have directed attention as strongly as they might have done to the influence of the oxygen contained in the saliva upon the digestive function. Here, as to a certain extent in the blood, the tribaric phosphate of soda seems to be the medium through which this gas is introduced. Arriving at the stomach it must quicken digestion, from its known properties. As it is, we cannot think that Wright's results have been materially altered by his successor, and certainly are of the opinion that Bernard's experiments need careful revision, and that his results must be received with great caution, contradicted as many of them are, by such able, zealous and conscientious experimenters.

*** The writer desires to acknowledge his indebtedness, in the preparation of this article, to Dr. Wright's papers in the Lancet for 1841 and '42; Drs. Marshall and Garrod's experiments, recorded in the same journal; G. Owen Rees' article on Saliva in the Cyclopœdia of Anatomy and Physiology; Simon's Chemistry of Man; the article on Lehmann's Physiological Chemistry in the British and Foreign Medico Chirurgical Review for October, 1851; Donaldson's paper on Bernard's recent discoveries in the American Journal of Medical Sciences for October, 1851, and the last edition of Carpenter's Elements of Human Physiology.

